# The Factors of National Health

**Published:** 1896-02-29

**Authors:** 


### The Hospital

An Institutional Journal of

The Medical Sciences and Hospital Administration, with

AN EXTRA NURSING SUPPLEMENT.

Vol. XIX.—No. 492.] [Feb. 29, 1896.

### The Factors of National Health.

Medical men are masters of detail. It is imperative that they should be so. Nevertheless, it is necessary, in forming a judgment, not only to see a thing "clearly," but also to see it "whole." The individual medical man concerns himself with his own patients; the whole body of medical men recognise the high duty of conserving and improving the health of the whole nation. At the present moment the health of the nation is a pre-eminently anxious question. A few years ago it was common to speak of Turkey as the "sick man." Everybody knew what was meant by that. In *esprit de corps*, in governing capacity, in finance, the empire of the Sultan seemed to be on the verge of dissolution. Is Great Britain, at the present moment, in all or in any senses "a sick man"?

The present time is opportune for a brief study of the nation from a physiological standpoint. Where do we stand as compared with our forefathers and with other nations in inherited physical and mental vigour, in national health habits, in courage, and in forethought and high purpose? No individual man can face great responsibilities who has not an inherited fund of physical and mental capacity, habits which make for health, a great spirit, and a high and steadfast purpose. As it is with the individual, so it is with the nation; a parallel exact in every important particular holds between a single man and the nation of which he is a part.

We are a nation against the world for the moment. What is our competence? A physiological history of the British people from the time of the Roman occupation would be profoundly interesting in this connection. The mixture of high and capable races, the comparative absence of intermarriages, the continued importations of new blood from Denmark and Norway, from the countries inhabited by the Saxons and Angles, from Normandy and France, and, indeed, from the strongest stocks to be found in all Western Europe, combined to form a race bold, hardy, persistent, resolute—a race fitted not merely to till the ground and fight defensive battles, but to spread itself out in all directions, conquering, ruling, subduing nature to the service of man, and establishing order and peace everywhere. By heredity the British are a physically strong and mentally resolute and persistent race. They have inherited that which makes a tame and submissive life impossible to them. Practically every man among us is a possible coloniser, a possible ruler wherever he may choose to establish himself, and a possible founder of a new order of things. All this is to be taken into account. Other nations would do well to realise it. We ourselves must realise it, and must accept our place in nature, and what is apparently our manifest destiny. Heredity is one of the most important of all the factors of national health.

After heredity comes the question of habits, ways of living. The strongest born man in the world must live physiologically or he cannot continue healthy and capable for his full life term. Exactly so is it with a nation. What are our national habits? Admirable, almost everything that could be wished when viewed as a whole. The school life of our boys makes men of them in all the essentials of physique, courage, and a saving self-respect. Not only is this so with our public school boys, but every year the boy of the Board school and the voluntary school tends to be fashioned more and more after the highest type of schoolboy known in the world. All this is of the primest importance in prognosticating the greatness and permanence of the English-speaking races. When the life of school is over our boys do not become prematurely middle-aged men, fitted into narrow grooves, and incapable of what may be called, in the language of zoology, a free-swimming existence. They remain young, plastic, high-spirited. They spend their leisure, like the professional athletes in the older States, in out-door games, venturous sports, strength-testing and strength-making amusements: so that at any moment in our country there are hundreds of thousands of young men in the finest possible physical condition, capable of unlimited fatigue, whom a single fortnight's training would render fit for a long and arduous campaign. The habits of the British people in respect of food and drink, in respect of occupations and amusements, and in respect of that equally important factor moral cleanliness, are beyond all example the habits of a strong, capable, enduring, unconquerable race.

Courage is one of the most indispensable of all the factors of national health and longevity. Now courage, in ultimate analysis, is a matter of blood-pressure, of cerebral blood-pressure. From very ancient times it has been usual to speak of a brave man as a man of "stout heart." How literally this is the case scientific physiologists alone know. Given a heart physically robust, and as the result a vigorous blood-pressure in the brain, it is almost impossible for its owner to be anything but a strong and enduring fighter. The Englishman is a substantial feeder. Even in modern times and in large towns he still preserves his splendid admiration for "roast beef" and all that it connotes. His present outdoor habits of cricket, football, golf, cycling—we are credibly informed that more than a million cycles were made in England last year—hunting, shooting, and the like, all tend to preserve his love of roast beef and his stomach's capacity for dealing with it. There is thus the best of all possible guarantees for a continuance of such a natural "stout heart" as will produce a natural vigorous blood pressure, and in consequence a national courage which must be practically invincible.

